# An Embedded Framework for Fully Autonomous Object Manipulation in Robotic-Empowered Assisted Living

**DOI:** 10.3390/s23010103

**Published:** 2022-12-22

**Authors:** Giovanni Mezzina, Daniela De Venuto

**Affiliations:** Department of Electrical and Information Engineering, Politecnico di Bari, 70125 Bari, Italy

**Keywords:** object manipulation, robotics, personal care robots

## Abstract

Most of the humanoid social robots currently diffused are designed only for verbal and animated interactions with users, and despite being equipped with two upper arms for interactive animation, they lack object manipulation capabilities. In this paper, we propose the MONOCULAR (eMbeddable autONomous ObjeCt manipULAtion Routines) framework, which implements a set of routines to add manipulation functionalities to social robots by exploiting the functional data fusion of two RGB cameras and a 3D depth sensor placed in the head frame. The framework is designed to: (i) localize specific objects to be manipulated via RGB cameras; (ii) define the characteristics of the shelf on which they are placed; and (iii) autonomously adapt approach and manipulation routines to avoid collisions and maximize grabbing accuracy. To localize the item on the shelf, MONOCULAR exploits an embeddable version of the You Only Look Once (YOLO) object detector. The RGB camera outcomes are also used to estimate the height of the shelf using an edge-detecting algorithm. Based on the item’s position and the estimated shelf height, MONOCULAR is designed to select between two possible routines that dynamically optimize the approach and object manipulation parameters according to the real-time analysis of RGB and 3D sensor frames. These two routines are optimized for a central or lateral approach to objects on a shelf. The MONOCULAR procedures are designed to be fully automatic, intrinsically protecting sensitive users’ data and stored home or hospital maps. MONOCULAR was optimized for Pepper by SoftBank Robotics. To characterize the proposed system, a case study in which Pepper is used as a drug delivery operator is proposed. The case study is divided into: (i) pharmaceutical package search; (ii) object approach and manipulation; and (iii) delivery operations. Experimental data showed that object manipulation routines for laterally placed objects achieves a best grabbing success rate of 96%, while the routine for centrally placed objects can reach 97% for a wide range of different shelf heights. Finally, a proof of concept is proposed here to demonstrate the applicability of the MONOCULAR framework in a real-life scenario.

## 1. Introduction

The automation of object and materials handling activities has been shown to have a high impact on efficiency and productivity increment in several different applicative areas spanning from healthcare to assistive ones, where most of these operations are still performed manually. For instance, a survey conducted in US and European hospitals [1] in 2018 observed that about 40% of the nurses’ time is dedicated to no-value-adding activities that involve repetitive delivery and retrieval of food trays, correction of erroneous drugs delivery, and so on [2]. The advances in robotics technology, such as the high number of sensors equipped on the automatons, the increasingly powerful on-board computers, the artificial intelligence-based algorithms, and the even more performant simultaneous source localization and mapping (SLAM) approaches, paved the way to the introduction of Autonomous Mobile Robots (AMRs) [3]. Due to their flexibility, AMRs can help medical staff, reducing their workload by carrying out easy and repetitive tasks such as food delivery or drug administration, improving—as a consequence—the efficiency of the overall healthcare structure (both in hospitals and in a domestic assistance framework).

In this context, several state of the art AMRs have been proposed to improve hospital and domestic logistics [1,2,3,4]. Some examples of applications are the automatic wagons used to move sterile instruments into the hospital area, or the automatic and secured cancer medicine transportation wagon, both proposed by Mobile Industrial Robots [3,5]. ABB also proposed an AMR to support medical staff in repetitive laboratory tasks, e.g., loading and unloading centrifuges and so on [3,6]. AMRs have also been employed for environment disinfection as proposed by UVD Robots in [3,7]. Focusing the review on those robotic platforms with arms and grippers, Diligent Robotics proposed Moxi. It is designed to solve some logistic tasks in the hospital, e.g., delivering medical samples, and carrying laundry [8]. Despite these capabilities, Moxi has not been empowered with human-interaction capabilities and is categorized as a service robot. Its use as a nurse robot to provide pills to patients has been avoided because patients felt uncomfortable with it. Also, PAL robotics were proposed [9] in some embryonal healthcare applications. Nevertheless, TIAGo uses a Robot Operating System (ROS) framework, which is not globally accepted for applications that treat sensitive data and for which full offline automation is needed [10].

Another category of widely used AMRs in healthcare facilities and domestic assistance is the social interactive robots. These kinds of robots are mainly designed for verbal and animated interactions with users and patients. Indeed, their main use in hospitals concerns reception tasks. This kind of robot did not find practical application in an assistive context, because most of the time they have two upper arms, but they lack object manipulation capabilities [1,3,11]. The most worldwide diffused socially interactive robot is Pepper by SoftBank Robotics (with 12 thousand units, only in Europe) [11]. Pepper has been typically employed as a receptionist in healthcare facilities; however, it was recently used to interact with people affected by dementia [12], and in post-stroke rehabilitation [13]. Despite its large use, the presence of a single servomotor to control the entire hand, and the absence of sensing feedback for force management, prevents its use in an assistive and support context for medical staff.

In this paper, we focus on expanding the capabilities of socially interactive robots by adding to them a set of automatic object manipulation routines. Due to their large diffusion and employment in healthcare facilities, the proposed functional re-design aims to make social robots also suitable for medical staff support and patient assistance both in domestic and ambulatory frameworks.

In this respect, we present the MONOCULAR framework (eMbeddable autONomous ObjeCt manipULAtion Routines), which embeds a set of manipulation routines based on the functional combination of data from two RGB cameras and a 3D depth sensor for reliable object grabbing. The MONOCULAR framework is designed to provide the robot with a stable object recognition system (i.e., pharmaceutical products in this application), approach them, and grab and scan them for check purposes. The MONOCULAR object recognition subsystem is based on an embedded version of the real-time object detection engine “You Only Look Once” (YOLO) by [14], and the Mini-YOLOv3 [15], which analyzes RGB frames streaming. By using this streaming, MONOCULAR is also used to estimate shelf characteristics (i.e., height), in order to autonomously drive its joint servomotors to adapt the grabbing procedure, without the need for internet connections or external frameworks like ROS. Movement and collision prevention is supported by depth mapping extracted via a single 3D sensor. To improve grabbing accuracy, MONOCULAR is designed to select the best routines according to the recognized object’s position. One of the MONOCULAR routines optimizes the navigation and body segments trajectories to approach and grab laterally placed objects. This routine is mainly inspired by our previous work [16]. Another implemented routine adjusts the robot’s operations in the presence of centrally placed objects.

Due to its wide use in healthcare facilities and domestic assistance frameworks, the Pepper robot was selected as the target platform to embed, characterize, and test the MONOCULAR framework in a real-life assistive scenario.

Ultimately, the breakthrough points of the proposed framework can be summarized as follows:

- To the best of our knowledge, MONOCULAR is a first-of-a-kind framework that embeds manipulation capabilities in socially interactive robots, addressing the technological limitations of the grippers (e.g., a single servomotor to manage the hand, no force feedback). This feature allows social robots to also be used in a more comprehensive care setting, given their widespread use in healthcare facilities.
- The proposed framework exploits on-board equipment for trajectory calculation and manipulation procedures. The system, therefore, does not require any additional equipment to be included in the environment. This choice permits us to minimize the cost for a potential robot upgrade and allows a quick relocation of the robot to various environments.
- As previously reported, the framework incorporates a routine already developed in our previous work [16]. In the context of this paper, MONOCULAR improves both this routine [16] and the one related to centrally placed objects, by dynamically adapting the grabbing systems to different shelf heights. This feature allows dynamic reprogramming of the approach parameters, overcoming the main problem in [16], i.e., following the fixed-height shelf approach. The introduction of two routines also makes it possible to increase the number of items that can be stored on the (variable-height) shelf, which will no longer be limited to those laterally placed.
- The presented system is designed to manipulate objects located on fixed positioning grids. However, the dynamic management of the driving parameters of the body segments was optimized to accommodate human errors in object positioning.

The paper is organized as follows. Section 2 outlines the MONOCULAR framework in terms of the hardware used and the manipulation routines. Section 3 provides the experimental results and a proof of concept. Finally, Section 4 concludes the paper.

## 2. The MONOCULAR Architecture

### 2.1. Hardware Platform

The MONOCULAR framework was embedded, optimized, characterized, and tested on a Pepper Y20 model by SoftBank Robotics (SoftBank Robotics, Tokyo, Japan).

Pepper is supplied with an Intel Atom E3845 (quad-core) 1.91 GHz processor, 4 GB DDR3 (2 GB dedicated to Operating System (OS)) of RAM, 8 GB eMMC (not available to user), and 16 GB of micro SDHC. All the routines proposed in the following execute on Pepper’s native operating system, NAOqi OS, which is a Gentoo-based GNU/Linux distribution [10]. For the sake of readability, only the details related to the sensors that are involved in the routines proposed here will be described in the following.

**Figure 1:**
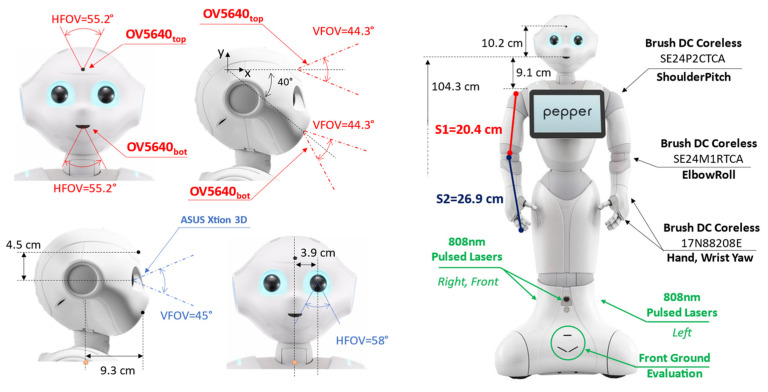
Hardware Platform Overview.

Pepper is provided with two RGB cameras and a 3D depth sensor as per Figure 1. Both RGB cameras are OV5640 (OmniVision Technologies Inc., Santa Clara, CA, USA). The first one, identified as OV5640top is placed on the forehead with an angle of 90° with respect to the head frame axis (0° considering the X-axis reference in Figure 1). The second camera, labeled OV5640bot, is placed on the robot mouth with an orientation of −40° with respect to the X-axis reference in Figure 1. The OV5640 provides a maximum resolution of 5 Mp with 55.2° of the horizontal field of view (FOV), 44.3° of vertical FOV, and a diagonal FOV of 68.2°. The output frames are set to kQVGA (320 × 240 px) with 5 fps.

Pepper is also provided with an ASUS Xtion 3D (ASUSTeK Computer Inc., Taipei, Taiwan) sensor, located in the left eye of the robot, with 0.3 Mp of resolution. The Xtion provides 58° of horizontal FOV, 45° of vertical FOV, and a diagonal FOV of 70°. The depth camera output was set to be kQVGA (320 × 240 px) with 5 fps.

The navigation routines, for the object being approached, also involve motor management to move the arm segments, reported in Figure 1 only for the right side as S1 and S2. Specifically, the routines manage six different motors for the different robot sections: head, arms, and hands. A brush DC coreless SE24PCTCA is driven for the head yaw and the shoulder pitch. A SE24M1RTCA is used to drive the head pitch and the elbow roll. Finally, the hands section is managed by two 17N88208E for the wrist yaw and the hand open and close. For navigation purposes, Pepper is also supplied with three holonomic wheels (spheric shape) placed at the base of the automaton.

For navigation purposes, Pepper is also equipped with six laser line generators emitting at 808 nm with a framerate of 6.25 Hz per laser. As per Figure 1, three main laser scanners are pointing toward the front, right, and left directions. Each laser scanner is composed of 15 laser beams able to update the directive distance value in ~160 ms. According to the constructor directives, the Horizontal Field of View (HFOV) of each laser scanner is 60°, while the Vertical Field of View (VFOV) is 40°. This leads to an overall HFOV of 240° with 60° of blind angle equally distributed across 45° and −45°. There are also three laser scanners used for front-ground evaluation. The maximum distance of detection is defined as up to 10 m with a maximum height of 10 cm for the objects that the lasers can detect.

### 2.2. MONOCULAR Working Principle: Preparation Steps

**Figure 2:**
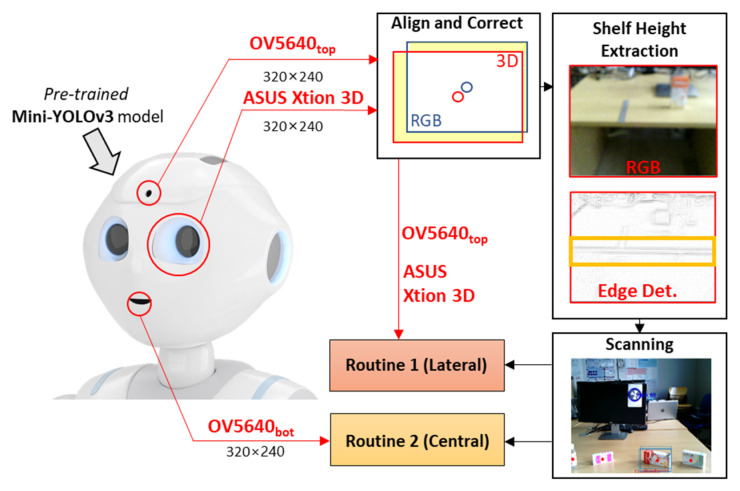
Hardware Platform Overview.

Figure 2 reports a schematic overview of the MONOCULAR framework. The workflow starts with the acquisition of a sequence of frames from the embedded top RGB camera (the forehead one) and the depth map frame from the 3D sensor placed in the left eye of the robot.

**Align and Correct.** The frames from the top RGB camera and the 3D sensors are displaced between them due to the mutual positioning and the dedicated field of view (FOV) as per Figure 1. For this aim, a dedicated step for image alignment and a correction step were needed.

The procedure is composed of two main steps as proposed in our previous work [16], i.e., (i) frame border correction, and (ii) frame extraction. Frame border correction operation consists of computing the FOV coverage of the top RGB camera (i.e., OV5640_top_—Figure 1) and the 3D sensor (i.e., ASUS Xtion 3D—Figure 1). During this step, the frames from the two sources are overlapped and the parts that are not shared are removed from the analyzed frame. This cropping operation is named frame extraction. According to [16], for a Pepper Y20 with the above-mentioned settings, 13 pixels should be removed on the top and 7 pixels on the right of the RGB frame.

Similarly, 13 pixels should be cropped on the left 3D sensor frame. Next, the cropped frames are resized (via bilinear interpolation) to match the closest multiple of 32 on both dimensions due to YOLO input constraint [17].

**Shelf Height Extraction.** To properly adapt both manipulation routines, the proposed system must estimate, as precisely as possible, the height of the shelf on which the objects to be grabbed are placed. For this purpose, MONOCULAR embeds a dedicated processing step that exploits frames from the top RGB camera and the 3D sensor.

**Figure 3:**
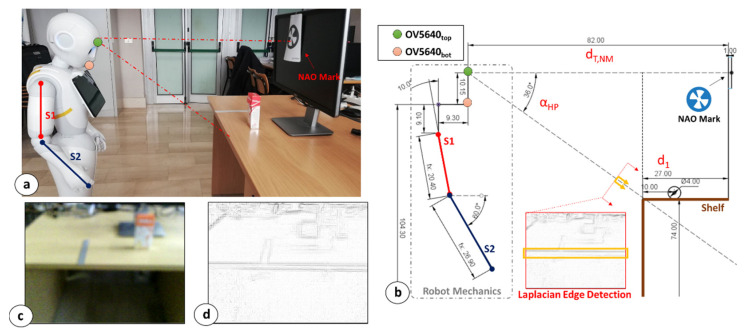
Shelf Height Extraction procedure. (**a**) Snapshot of the experimental setup; (**b**) Robot mechanics explanation for the procedure; (**c**) RGB camera snapshot; (**d**) Laplacian Edge detector outcome for the frames in (**c**).

Figure 3a shows the height estimation setup. The first step of this procedure consists of acquiring the distance between the 3D camera coordinates and the center point of an NAO mark placed on a frontal surface via depth map data. This distance is named d_T,NM_ in Figure 3b. To maximize the accuracy of the estimation, the NAO mark should be roughly placed at the same height of the 3D sensor.

Once d_T,NM_ is extracted, the pitch value for the head frame is increased up to α_HP_ = 36° (max allowed: 36.5°). Next, MONOCULAR queries the robot for a top RGB camera photo, which is analyzed via an embedded and low-complexity edge detection algorithm considering a 3 × 3 convolution mask [18]. Specifically, an edge detection with a 2nd derivative using a Laplacian of Gaussian (LoG) filter and zero-crossing (with σ = 3 for the Laplacian of Gaussian kernel) was implemented as Python script on NAOqi. Figure 3d shows an example of the LoG filtering application starting from the camera frame in Figure 3c.

If the central area of the resulting image (Figure 3d) is occupied for >70% by edge pixels, it means that the shelf edge is centered with the FOV of the camera. If <70% is detected, the robot starts moving toward the shelf with preset steps. Optimal positioning is reported in Figure 3a,b, where the center of the RGB camera frame coincides with the shelf edge. In the proposed application, the central area is defined as the whole frame width (i.e., 320) and a restricted area of the frame height that corresponds to the range of pixels 120 ± n_px_, where n_px_ are the limits of the area (n_px_ = 15 pixels in the proposed application).

Since the distance between the NAO mark and the shelf edge is known a priori (d_1_ in Figure 3b), the height of the shelf can be estimated as per the following equation:

(1) $$h_{\mathrm{sh}}=h_{\mathrm{ct}}-\left(d_{T,\mathrm{NM}}-d_{1}\right)\cdot \mathrm{tg}\left(\alpha_{\mathrm{HP}}\right)$$

where $h_{\mathrm{sh}}$ is the shelf height, $h_{\mathrm{ct}}$ is the height of the top RGB camera (i.e., 114.45 cm), $d_{T,\mathrm{NM}}$ is the distance between the camera and the NAO mark as per Figure 3b, while $\alpha_{\mathrm{HP}}$ is the head pitch angle. The distance between the NAO mark and the shelf edge, $d_{1}$, is preset due to the employment of a positioning grid that allows the placement of the object only in specific areas.

**Scanning.** Once the shelf height is estimated, the cropped and resized RGB acquisition is then sent to an object detection routine based on the Mini-YOLOv3 method [14,15]. The Mini-YOLOv3 model must be pretrained offline to recognize specific objects. Generally, the YOLO method oversees extracting a number of bounding boxes returning; for each of these, the probability that the box contains an object and the probability that the object belongs to a specific class. Since all the objects are placed at specific coordinates on the shelf, the MONOCULAR framework decides the manipulation routine to be carried out. It can be Routine 1 if the objects are laterally placed from the robot’s point of view, or Routine 2 if the object is centrally placed. Routine 1 involves the top RGB camera and the 3D sensor, while Routine 2 mainly involves frames from the bottom RGB camera. Routine 1’s design idea was investigated in our previous work [16], but no considerations about the shelf height, object characteristics and dynamic parameters’ adaption were provided.

### 2.3. MONOCULAR Working Principle: Object Detection

The MONOCULAR object detection engine was entrusted to the portable version of YOLOv3, the Mini-YOLOv3 by [15]. Mini-YOLOv3 proposes a lightening of the YOLOv3 backbone network (i.e., darknet-53), employing only 16% of the initial parameters. This reduction led to the degradation of the network’s accuracy. To improve the performance of multi-scale object detection, a Multi-Scale Feature Pyramid network based on a simple U-shaped structure was employed according to [15]. The above-mentioned structures (lightweight backbone and Multi-Scale Feature Pyramid network) are used to extract the features maps from the input image, and to produce bounding boxes based on the learned features. Specifically, the Multi-Scale Feature Pyramid network is composed of three main parts: (i) the Concatenation module; (ii) the Encoder-Decoder module; and (iii) the Feature Fusion module. The Concatenation module is used to connect each feature generated by the backbone network and the Encoder-Decoder module is used to generate multi-scale features. The Feature Fusion module is used to aggregate features for the final prediction. The Non-Maximum Suppression (NMS) approach is used to produce the classification results.

This approach was selected due to the optimal trade-off between two main discrimination metrics for the object detection algorithms: the number of floating-point operations (FLOPs) and the mean average precision with Intersection over Union (IoU) of 0.5, typically identified as mAP@0.5, as declared by dedicated works considering a standard dataset (i.e., COCO test-dev2017) [15,17]. The object detection speed, in frames per second (FPS), was excluded by this analysis due to the RGB camera setting for the streaming speed (i.e., 5 fps). Moreover, only object detection algorithms that provide the weights of the backbone network were considered for further investigation. This constraint was needed due to the limited number of images related to the object to be classified for the proof of concept. As per the previous constraints, the employment of a four object detection algorithm (i.e., Mini-YOLOv3, YOLOv3-tiny, YOLOv3- tiny pruned and YOLOv4-tiny) was investigated. According to data reported in [15,17], Mini-YOLOv3 achieves the highest mAP@0.5, even executing a high number of FLOPs with respect to other solutions. Indeed, Mini-YOLO executes 10.81 billion FLOPs for an mAP of 52.1%, YOLOv3-tiny employs 5.57 billion FLOPs for an mAP of 33.1%, 3.47 billion FLOPs are executed by YOLOv3-tiny pruned with an mAP of 33.1% and, finally, 6.91 billion FLOPs and an mAP of 40.2% are the parameters of the YOLOv4-tiny.

The object detection model pre-training was carried out offline and consisted of data collection and preparation, model training, inference testing, and model extraction. During the data collection, images were captured both via the forehead and mouth camera, with different operative angles (repeating the approach routine every time).

Four classes of pharmaceutical packages were considered for the proof of concept: *{Fluifort, Aciclovir, Antipyretic, Hand Sanitizer}*. Overall, 2496 images were collected for a total of 9984 annotations (4 per image). The labeling was manually realized via Labellmg software. As per the guidelines in [16], during the labeling, the bounding boxes were drawn including the entirety of the object and a small amount of space between the object and the bounding box. Extracted data were further pre-processed via a Roboflow framework to implement data augmentation and auto-orienting on the gathered data [19]. The above-mentioned preparation steps were carried out on Google Colab. The same online notebook was employed for the Mini-YOLOv3 implementation via Keras with TensorFlow backend, running a repetitive fine-tuning approach, by freezing and unfreezing the backbone body. Specifically, new data were uploaded via the Roboflow setting with a batch size of 32, epochs at 500, and an Adam optimizer with a learning rate of 10^−3^ with the reduced darknet body full-freeze and 10^−4^ for unfreezing and fine-tuning. The fitted model, with the custom weights, was exported as a .py script on NAOqi OS and implemented on the Pepper robot.

### 2.4. MONOCULAR Working Principle: Routine 1

The MONOCULAR Routine 1 concerns the laterally placed objects according to [16]. Briefly, it consists of two main steps: (i) Tag Extraction, and (ii) Object-vs.-Hand Coverage Routine.

**Tag Extraction.** During this step, the robot moves towards the object by keeping the selected YOLO tag in the center of the image and progressively adjusting the head frame.

**Figure 4:**
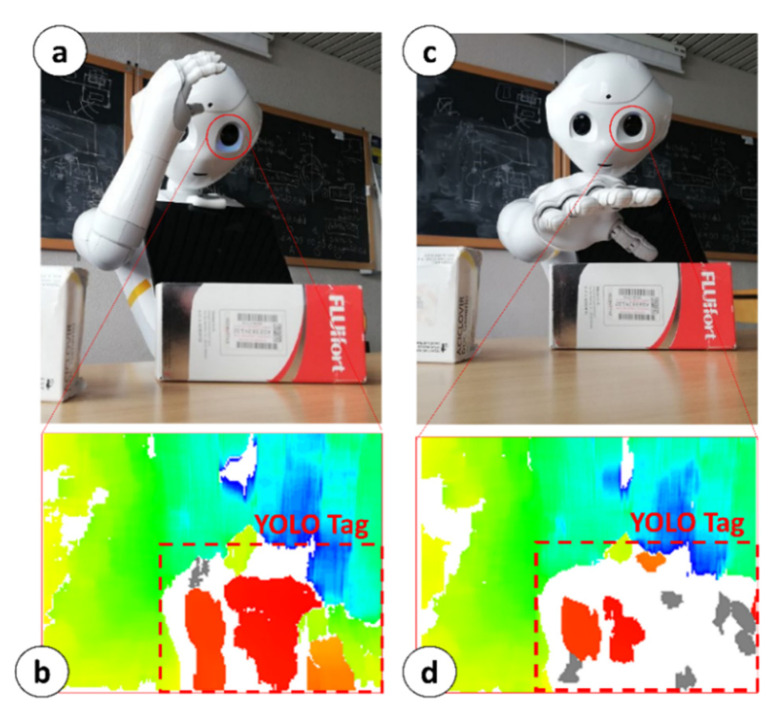
MONOCULAR Routine 1: Object-vs.-Hand Coverage Routine. (**a**) Object approaching step; (**b**) Depth map for the extraction of initial number of pixels; (**c**) Object-vs.-Hand Coverage; (**d**) Depth map for the extraction of coverage percentage.

According to the object’s position with respect to the robot’s torso frame, MONOCULAR selects the arm to be used for grabbing and manipulation. During the object’s approach, the arm selected for grabbing is led to the position shown in Figure 4a. The aligned RGB image from the top camera and the 3D image are used to extract the area covered by the YOLO tag (Figure 4b) to proceed with further analysis. The central anchor point of the YOLO Tag is used to define a reference distance from the depth map of the 3D sensor.

This reference distance is used to fix a reference useful to calibrate the segmentation blob during the 3D reconstruction as per Figure 4b. This reference can be identified in Figure 4b as a red blob. The depth threshold for the segmentation is set to 5 mm, and the colormap limits are restricted in the proximity of the reference value. Figure 4b shows the initial blob vision from Position #1. The area included in the red blob determines 100% of the uncovered object. MONOCULAR stores the number of “red pixels” from the depth map.

**Object-vs.-Hand Coverage Routine**. Once the initial number of pixels is set, the hand moves onto the selected object, partially covering it, from the point of view of the 3D sensor (see Figure 4c). MONOCULAR evaluates the difference between the initial number of red pixels, and the final one (after moving the hand). If the difference falls within an experimentally derived range (i.e., ~40–45% for right hand manipulation, 25–32% for left hand), the hand stops moving, and the grabbing routine starts running. More details about the Object-vs.-Hand Coverage Routine and its characterization are available in [16].

Once the robot’s hand is in the position shown by Figure 4c, the shoulder pitch is progressively adapted to press against the shelf plane to ensure a good grabbing force on the object. Since the shelf height, $h_{\mathrm{sh}}$, is derived from the Shelf Height Extraction step, MONOCULAR can extract a desired shoulder pitch angle to ensure it can extract the desired shoulder pitch, which ensures the correct pressure on the plane without overloading the mechanical shoulder joint. The actual degrees read by the shoulder motor are compared with the desired ones extracted by MONOCULAR. If the actual degrees stop decreasing, it means that the object has been blocked and no additional pressure needs to be applied, preserving the joint from overloading. This step ends with a full-hand closing.

The object is—finally—scanned through the image recognition engine to confirm the request matches. The robot is now ready to deliver the pharmaceutical envelope.

### 2.5. MONOCULAR Working Principle: Routine 2

Routine 2 of the MONOCULAR framework involves the objects that are placed centrally on the shelf. The procedure is realized by means of five main steps: (i) Target Height Extraction, (ii) Arms Positioning, (iii) Object Approaching, (iv) Hand Selection and Mutual Coverage, and (v) Grabbing and Scanning.

**Target Height Extraction.** This step starts with the assessment of the shelf height parameter, $h_{\mathrm{sh}}$, and the height of the object to be grabbed, namely $h_{\mathrm{obj}}$. This latter parameter is stored in the object dictionary accessible via Pepper’s memory recall.

As a first step, MONOCULAR estimates the maximum height that the whole arm, given by the combination of S1 and S2 segments with proper orientation, should have. For this purpose, a target height is extracted according to the equation:

(2) $$h_{\mathrm{tg}}=h_{\mathrm{sjnt}}-\left(h_{\mathrm{sh}}+\frac{h_{\mathrm{obj}}}{a}\right)$$

where $h_{\mathrm{tg}}$ is the target height defined as the maximum height that the arm (S1 + S2) should have to properly approach the object, $h_{\mathrm{sjnt}}$ is the shoulder joint height (i.e., 95.2 cm in this application), while a is a coefficient related to the height of the object, $h_{\mathrm{obj}}$. The coefficient a is provided by the dictionary of objects stored in the Pepper memory and is related to the mass distribution of the package. Experimental analysis showed that a general height of approach of $\frac{h_{\mathrm{obj}}}{2}$ (i.e., with a = 2) allows the robot to achieve high grabbing accuracy with most of the packages involved in the proof of concept.

**Figure 5:**
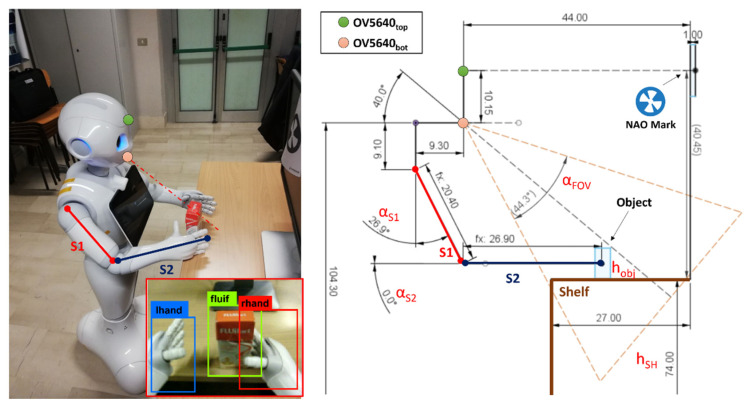
Arm Positioning and Object Approaching phases of MONOCULAR Routine 2 with robot mechanics explanation.

**Arms Positioning.** Once the $h_{\mathrm{tg}}$ is estimated, MONOCULAR compares $h_{\mathrm{tg}}$ with the length of the segment S1 ($l_{S1}\;$= 20.4 cm in our application). If $h_{\mathrm{tg}}\leq l_{S1}$, according to Figure 5, MONOCULAR computes the angle that the S1 segment should keep with respect to the torso axis ($\alpha_{S1}$ in Figure 5), according to the following equation:

(3) $$\alpha_{S1}=\cos^{-1}\left(\frac{h_{\mathrm{tg}}}{l_{S1}}\right)$$

In this configuration, the second segment, S2, remains perpendicular to the torso axis as per $\alpha_{S2}$ in Figure 5.

Nevertheless, if $h_{\mathrm{tg}}>l_{S1}$, the second segment S2 must be moved, by increasing $\alpha_{S2}$ in Figure 5 to compensate for the difference between $h_{\mathrm{tg}}$ and $l_{S1}$, according to the equation:

(4) $$\alpha_{S2}=\sin^{-1}\left(\frac{h_{\mathrm{tg}}-l_{S1}}{l_{S2}}\right)$$

where $l_{S2}$ represents the length of the second arm segment S2. The combination of $\alpha_{S1}$ and $\alpha_{S2}$ is mirrored on the opposite arm.

**Object Approaching.** Once Pepper assumes the correct posture in a safe area (with no collision), the MONOCULAR framework starts driving the robot toward the object, keeping the achieved grabbing position as per Figure 5. The movement considers the distance between the robot and the NAO mark (through 3D sensor) and the distance between the object and the shelf edge (preset).

All those approaching movements are carried out slowly to avoid its omnidirectional wheels slipping. In addition, every movement is controlled by a dedicated odometric algorithm to compensate for incomplete movement errors [20,21]. When in the grabbing position, MONOCULAR runs an object position check step. This consists of taking a frame of the bottom RGB camera perspective that is sent to YOLO for stable labeling.

As a first step, the proposed system extracts the distance between the camera and the object’s position, calculating the parameter d as follows:

(5) $$d=\left(l_{S1}\cdot \sin\left(\alpha_{S1}\right)+l_{S2}\cdot \cos\left(\alpha_{S2}\right)\right)-9.3$$

where 9.3 cm corresponds to the horizontal distance between the bottom camera and the shoulder joint position according to the robot mechanics in Figure 5. The projection of d on the bottom camera axis is given by:

(6) $$x=\frac{d}{\cos\left(40{}^{\circ}\right)}$$

where 40° is the mutual angle of the OV5640 cameras (bottom camera axis). Starting from the above-presented parameter, x, it is possible to define the vertical field of view of the camera.

This vertical field of the view projection is mathematically defined as:

(7) $${\mathrm{VFoV}}_{\mathrm{obj}}=2\cdot x\cdot \mathrm{tg}\left(\frac{{\mathrm{VFoV}}_{\mathrm{bot},\mathrm{cam}}}{2}\right)$$

where ${\mathrm{VFoV}}_{\mathrm{obj}}$ is the projection of the vertical field of view of the camera (${\mathrm{VFoV}}_{\mathrm{bot},\mathrm{cam}}$) in correspondence with the object. The extraction of the ${\mathrm{VFoV}}_{\mathrm{obj}}$ parameter allows MONOCULAR to assess the correct robot–object alignment.

For this purpose, the framework considers only the central vertical column of the taken frame (480 rows from the column with index 320). Then, it extracts the minimum number of pixels that should belong to the object in a specific zone of the frame. This zone depends on the object height, $h_{\mathrm{obj}}$, shelf height, $h_{\mathrm{SH}}$, and robot arms’ position.

Specifically, considering an object placed on the shelf, it is possible to derive the position of its base as:

(8) $${\mathrm{Ob}}_{\mathrm{low}}=h_{\mathrm{bot},\mathrm{cam}}-x\cdot \cos\left(50{}^{\circ}\right)-h_{\mathrm{SH}}$$

where $h_{\mathrm{bot},\mathrm{cam}}$ is the height of the bottom camera (i.e., 104.3 cm in this application), while 50° is derived by 90°–40° with 40° being the mutual angle of the OV5640 cameras.

The ${\mathrm{Ob}}_{\mathrm{low}}$ can be expressed in pixels considering the following relationship:

(9) $${\mathrm{Ob}}_{\mathrm{low}|\mathrm{pxl}}=240-\left\lceil \frac{{\mathrm{Ob}}_{\mathrm{low}}}{\left(\frac{{\mathrm{VFoV}}_{\mathrm{obj}}}{480}\right)}\right\rceil$$

MONOCULAR also extracts the upper limit starting from ${\mathrm{Ob}}_{\mathrm{low}|\mathrm{pxl}}$, as per:

(10) $${\mathrm{Ob}}_{\mathrm{high}|\mathrm{pxl}}={\mathrm{Ob}}_{\mathrm{low}|\mathrm{pxl}}+\left\lceil \frac{h_{obj}}{\left(\frac{{\mathrm{VFoV}}_{\mathrm{obj}}}{480}\right)}\right\rceil$$

If the desired object is recognized, at least, in the vertical range [${\mathrm{Ob}}_{\mathrm{low}|\mathrm{pxl}},{\;\mathrm{Ob}}_{\mathrm{high}|\mathrm{pxl}}$] as defined by Equations (9) and (10). The grabbing can start, otherwise, the robot should adjust its position to fit the range.

**Figure 6:**
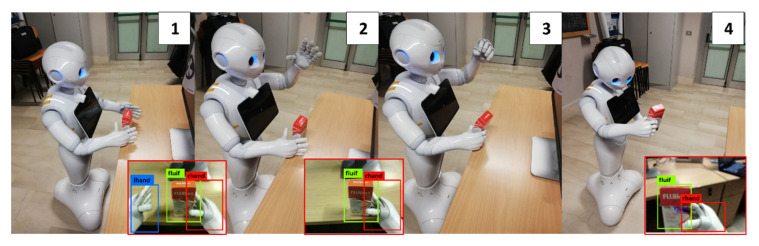
MONOCULAR Routine 2: four-frame demonstrative sequence for grabbing and scanning centrally placed objects. Frames are numbered from 1 to 4. Step 1: Object approaching, Step 2: Hand Selection and Mutual Coverage step with body adjustments. Step 3: Grabbing procedure. Step 4: Scanning.

**Hand Selection and Mutual Coverage.** When the object position check is completed (Figure 6—step 1), the MONOCULAR framework starts assessing the overlapping degree among the hands bounding boxes by YOLO and the recognized object to be grabbed.

The hand with the bounding box mostly overlapped with the object is selected for the grabbing procedure, while the other hand is raised to avoid interference in lateral adjusting movements (Figure 6—step 2).

If the overlap involves less than 25% of the hand box, the robot is laterally moved to cover at least this limit.

**Grabbing and Scanning.** Once the alignment is over the object, MONOCULAR commands the hand to close (Figure 6—step 3). The routine ends with scanning, which is carried out utilizing the image recognition system.

## 3. Experimental Results

This section aims to provide an overview of the manipulation capabilities introduced, via the MONOCULAR framework, on the Pepper robot by SoftBank Robotics. All the MONOCULAR algorithms and the procedure have been implemented as Python scripts on NAOqi OS and executed in the background.

To assess the achieved capabilities and to permit the robot’s application in a real-life scenario, a total of 2490 approaching, detection, and grabbing operations were conducted to characterize Routine 1 of MONOCULAR. Moreover, a total of 568 operations were carried out to characterize Routine 2.

### 3.1. Routines 1 and 2: Distance for Object Detection

The first analysis carried out for both routines was the identification of the maximum distance to ensure stable object detection via YOLO. To characterize this distance, two parameters were extracted: (i) the approach distance, and (ii) the tag extraction. The first distance returns a measure of the maximum distance needed to recognize “from afar” the needed object. In this situation, the robot system builds a first bounding box useful for the scanning procedure shared among the two manipulation routines embedded in the MONOCULAR framework. The tag extraction distance concerns the range of distances used to extract the bounding box via YOLO for both the hand-vs.-object coverage routine (Routine 1) and the object approaching step (Routine 2).

Let us define a dedicated parameter named stable object recognition accuracy (SORA). The SORA parameter is defined as the number of times the YOLO bounding boxes appear over the 10 analyzed frames (~2 s) at a specific distance. A total of 10 frames out of 10 with a YOLO bounding box leads to a SORA = 100%, 9 out of 10 SORA = 90% and so on).

Seven approaching distances were evaluated in the range 1–2.5 m (step 0.25 m), because above 2.5 m, no stable measurements were recorded, and because choosing distances below 1 m invalidates the approach’s usefulness. Ten runs per distance step contributed to the dedicated dataset, which contains data from a total of 70 approaching operations. The selected shelf height, $h_{\mathrm{sh}}$, was 74 cm.

The experimental results show that, with a distance of 1.25 m, the SORA approaches 84 ± 12.64%. Nevertheless, the range between 1 m and 1.75 m permits a SORA above 80%, with no strict constraints. It ensures good flexibility to potential odometry errors in navigation.

Experimental results concerning the SORA parameter versus the analyzed tag extraction distances were carried out considering six steps of distance in the range 35–40 cm (step 1 cm as per the odometry control resolution). Ten runs per distance step were considered, for a total of 60 approaching routines. Results show that above 37 cm, the SORA starts to strongly decrease. The reason lies in the data collection procedure for the YOLO training. Most of the collected images were acquired with tag extraction distances of 35 ± 4 cm. All of the above-proposed routines exploited the proposed optimal distances.

### 3.2. Routine 1 and 2: Shelf Height Range

The present section aims to characterize the maximum and minimum shelf height that ensures a good grabbing success rate for the MONOCULAR framework. For this purpose, a package was placed in its nominal position on a shelf with a variable height parameter. The shelf height was chosen to be compatible with the target height, $h_{\mathrm{tg}}$, to be tested. In this respect, fourteen different $h_{\mathrm{sh}}$ were tested, ranging from 64 cm to 90 cm with a step of 2 cm. For every height value, 10 grabbing attempts were carried out.

In this context, the Grabbing Accuracy parameter was introduced. This parameter is defined as the success rate in the number of successfully picking up and scanning operations over the 10 runs (e.g., 1 grab out of 10 leads to a grabbing accuracy of 10%).

**Figure 7:**
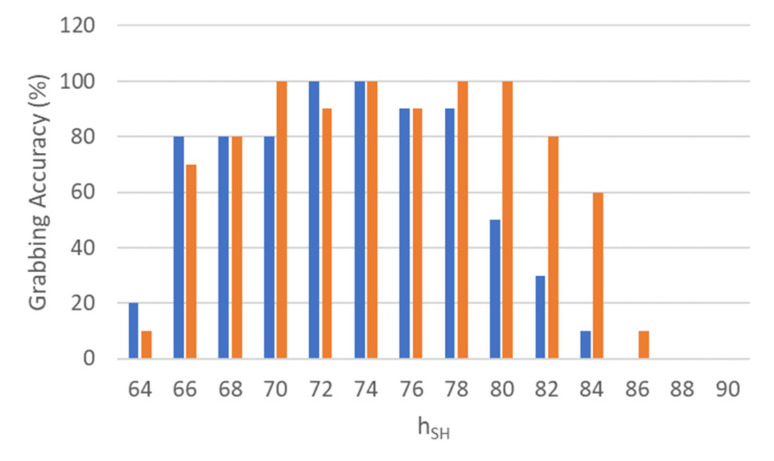
Grabbing accuracy of MONOCULAR Routine 1 (blue bars) and Routine 2 (red bars) versus shelf height ($h_{\mathrm{sh}}$).

Figure 7 summarizes the grabbing accuracy versus the shelf height, $h_{\mathrm{sh}}$, considering both of the analyzed routines.

Considering Routine 1 (blue bars), the results show that with shelf height included in a range that goes from 72 cm to 78 cm, the grabbing accuracy was ≥90%. Height values above 78 cm experienced a decrease in accuracy, given the inability of the shoulder joint to implement enough pressure toward the plane to allow for proper grip. Below 72 cm, the object-vs.-hand coverage routine starts modifying the dedicated coverage range values resulting in a decrement of grabbing accuracy.

Results concerning Routine 2 (red bars) show that with a shelf height within the range of 70–80 cm, the grabbing accuracy was constantly above 90%. Height values above 80 cm saw a drastic decrement in accuracy. This behavior is related to the increment of $\alpha_{S1}$ above 68°. In this respect, experimental analysis on the $\alpha_{S1}$ parameter demonstrated that the brush DC coreless SE24PCTCA of Pepper was not able to keep a reliable angle value above this limit. This limits MONOCULAR routine use above 84 cm. Another limitation of the routine above 84 cm concerns the object check routine because the object tended to disappear from the bottom camera’s field of view.

### 3.3. Routine 1: Object Positioning and Orientation

Concerning MONOCULAR Routine 1, an analysis concerning the optimal and suboptimal object positioning and orientation was carried out, in order to define a range in which it is more probable that a grabbing action ends successfully. For this purpose, a package—centered in its nominal position on the shelf (X0, Y0)—was rotated with the help of a protractor. Specifically, 37 angles ranging from 0° to 180° with 5° steps were assessed in terms of grabbing accuracy. Ten runs per angle were recorded.

The definition of grabbing accuracy provided in Section 3.2 was also employed in this context.

**Figure 8:**
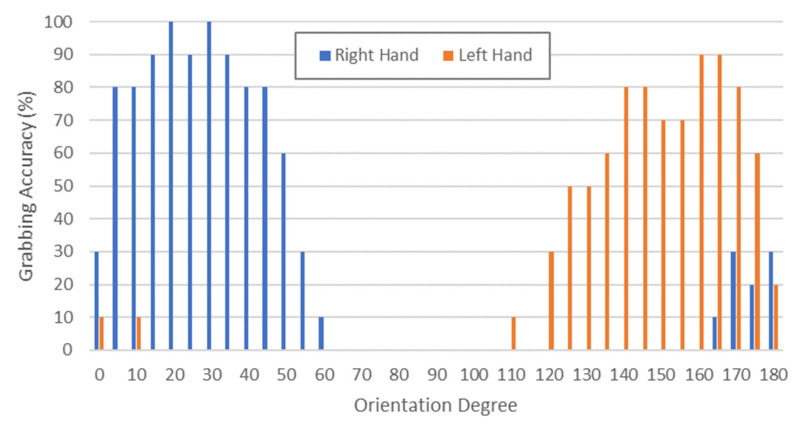
Grabbing accuracy of MONOCULAR Routine 1 with right hand (blue bars) and left hand (red bars) versus orientation degree.

Figure 8 shows the grabbing accuracy versus the orientation degree if the manipulation Routine 1 is carried out with the right (blue bars) or left (red bars) hand.

Results in Figure 8 show that, considering the right hand, a grabbing accuracy higher than 80% is ensured in the range 5–45°, with peaks in the range 15–35° (accuracy >90%).

A mirrored behavior was recorded for the left hand, which achieved its best performance in the range (180–10)° to (180–40)°.

The grabbing procedures also returned useful assessments concerning positioning tolerance. As discussed in [16], results showed that the package can be positioned with a tolerance of ±2.5 cm along the Y-axis for both hands and ±5.48 cm along the X-axis, without affecting the success rate. These results ensure that a minimal human error in the package’s positioning does not affect the grabbing success rate.

### 3.4. Routine 1: Hand-vs.-Object Coverage

As stated in Section 2.4, Routine 1 is based on a parameter known as the hand-vs.-object coverage percentage. This factor defines the number of pixels that remain uncovered by the hand, considering the FOV of the 3D sensor. This parameter was investigated in [16] via fifty runs per each considered coverage percentage. The analyzed range was 20% to 70% with a step of 2%. The experimental results showed that Routine 1 of the MONOCULAR framework requires a coverage between 40% and 45% if grabbing is carried out with the robot’s right hand, and 25–32% if the operations are realized via the left hand.

### 3.5. Routine 2: Grabbing Accuracy

To provide an overview of the grabbing capabilities obtained by adding Routine 2 into the MONOCULAR framework, 568 complete operations involving all five phases: (i) Target Height Extraction, (ii) Arms Positioning, (iii) Object Approaching, (iv) Hand Selection and Mutual Coverage, and (v) Grabbing and Scanning, were carried out.

**Figure 9:**
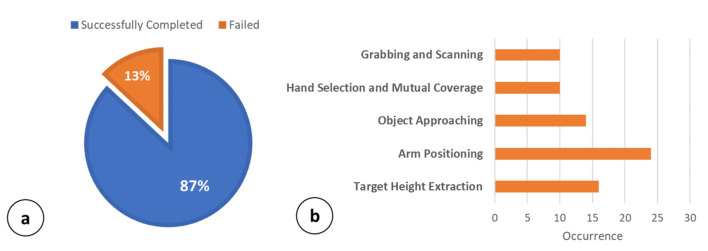
Grabbing accuracy of MONOCULAR Routine 2. (**a**) Success rate, (**b**) Error occurrence versus involved Routine 2 phase.

Figure 9a summarizes the grabbing success rate of the operations carried out. Results in Figure 9a show that, overall, 494 out of 568 operations (~87%) were successfully completed without any errors. However, in 13% (74 out of 568) of the cases, the operations failed due to errors of different natures. In this respect, Figure 7b summarizes the main failing causes, referring to the affected phases. Results show that in 16 out of 74 cases (21.6%), the operations were stopped due to erroneous target height estimation. Specifically, this error was mainly due to an error higher than 2 cm on the shelf height, $h_{\mathrm{sh}}$, estimation. The worst case concerns the Arm Positioning phase, with 24 failures out of 74 (32.4%). In this case, the operations were stopped due to a collision between the robot’s hand and the shelf (erroneous $h_{\mathrm{sh}}$) or $\alpha_{S1}$ and $\alpha_{S2}$ angle actuations.

**Figure 10:**
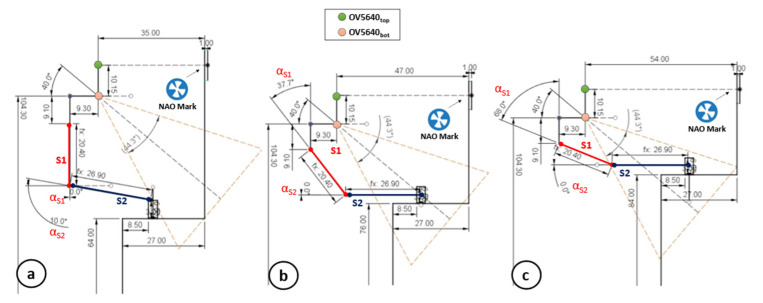
Robot mechanics in three main cases: (**a**) minimum shelf height; (**b**) typical shelf height; and (**c**) high shelf.

In 14 out of 74 cases (18%), the object approaching operations were not completed successfully. The object was mainly outside the range [${\mathrm{Ob}}_{\mathrm{low}|\mathrm{pxl}},{\;\mathrm{Ob}}_{\mathrm{high}|\mathrm{pxl}}$]. Only 10 failed operations (13.5%) concerned the Hand Selection and Mutual Coverage phase. Almost all of the failed operations showed an overlap percentage among the YOLO bounding boxes near the proposed limit of 25–27%. Finally, the Grabbing and Scanning procedure was the cause of 10 out of 74 failures (13.5%). In total, 6 out of 10 failures occurred due to a missing grab (object slipping), while 4 out of 10 were due to labels being missed by the image recognition engine. Below 70 cm, the main problems were related to the increment of $\alpha_{S2}$. When increasing $\alpha_{S2}$ above ~10°, the grabbing is not perpendicular and tends to be less effective with regards to the considered package. Figure 10 reports the robot mechanics in three main cases: (a) minimum shelf height (i.e., 64 cm); (b) typical shelf height (i.e., 76 cm); and (c) high shelf (i.e., 84 cm).

### 3.6. Routine 2: Bounding Boxes Overlap Percentage

To characterize the best bounding boxes overlap percentage for Routine 2 of the MONOCULAR framework, a package was placed in its nominal position on the shelf. Then, different bounding boxes overlap percentages were assessed in terms of grabbing accuracy. For the specific case, 11 different values of percentage were assessed, starting from 5% up to 55%. A step of 5% was chosen due to the difficulty in achieving a precise percentage value below this limit. In addition, above 55% in most of the cases, the package falls or starts to solidly shift with the hand, making the assessment useless.

**Figure 11:**
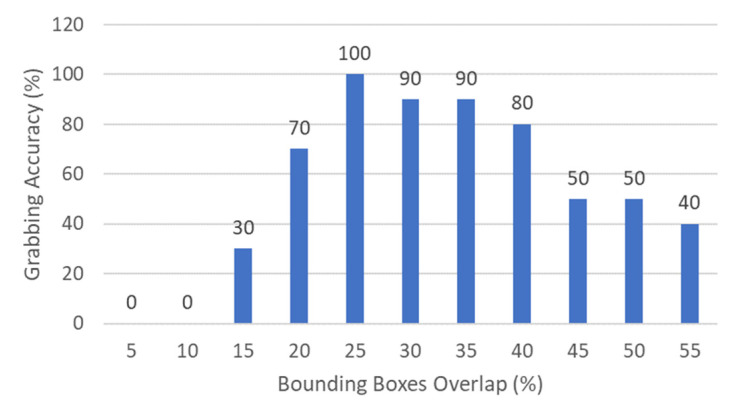
Grabbing accuracy of MONOCULAR Routine 2 versus bounding boxes overlap percentage.

For testing purposes, an $h_{\mathrm{sh}}$ of 76 cm was chosen. The arm segments were always set with the following angles: $\alpha_{S1}\;$= 37.7° and $\alpha_{S2}\;$= 0°. For every bounding box overlap percentage, ten identical runs were carried out. The experimental test results are shown in Figure 11.

Data from Figure 11 show that the best overlap range moves from 20% and 40%, with a peak of 25%. Above this limit, empty or semi-empty pharmaceutical packages start falling or shifting. This results in a reduction in grabbing accuracy with the increment of the overlap.

### 3.7. Routine 1 and 2: Scanning

The scanning step, shared by the MONOCULAR routines, constitutes the last stage of the proposed system. This step is responsible for recognizing objects to be delivered.

Therefore, to provide an inclusive overview of the functionality of this step, the sensitivity of the object recognition system will be analyzed in the following. This parameter (i.e., sensitivity) is defined as the ratio of true positives (TP) and positives (P). In the context of the present analysis, TP identifies those frames in which there is a correct identification of the grabbed object, while positives (P) are the total number of frames containing the object. Since the scanning procedure starts only if the wrist rotation is completed, and the object is positioned in front of the camera used for verification, the frames defined as P will constitute the entirety of the analyzed dataset.

Specifically, one hundred grabbing procedures were completed (25 for each of the items to be recognized). Each item was held in the scanning position for 10 s totaling 50 frames per grab, for a total of 500 frames (125 for each item).

Scanning results show that for pharmaceutical envelope #1 (Fluifort), 119 frames out of 125 were correctly classified (Sensitivity = 95.2%). The remaining six frames did not return a classification. Pharmaceutical product #2 (Aciclovir) returned correct classification in 95 frames out of 125 (Sensitivity = 76%). A wrong classification was recorded in 5 out of 30 frames. In these cases, the system classified the object as Antipyretic (pharmaceutical package #3). No classification was provided for 25 frames. Concerning envelope #3 (Antipyretic), 101 out of 125 frames were correctly classified (Sensitivity = 80.8%). In 4 out of 24 cases, the frames were labeled as product #2. Finally, object #4 (Hand Sanitizer) was correctly classified in 113 cases (Sensitivity = 90.4%). The remaining six frames did not return a classification. The results demonstrate that untexturized pharmaceutical envelopes (i.e., #2 and #3) are more prone to wrong classifications.

### 3.8. MONOCULAR: Proof of Concept in AMICO Project

**Figure 12:**
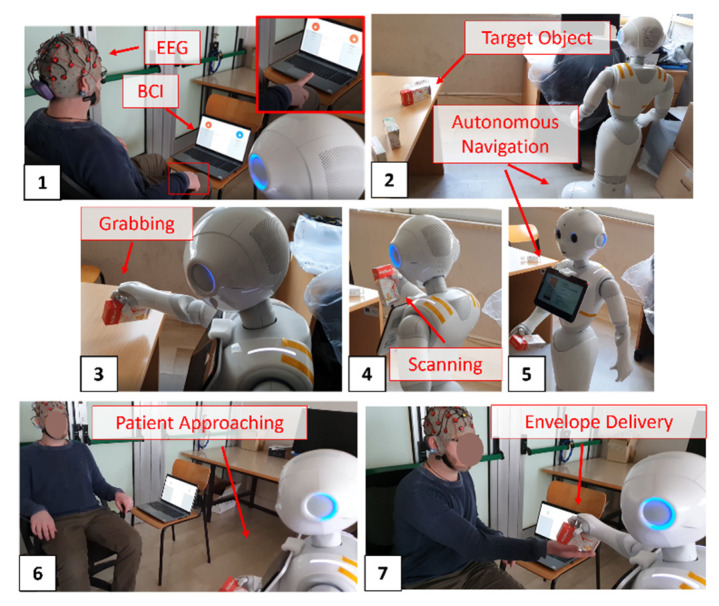
Proof of concept: Self-medication drugs request, pick-up and delivery. Step 1: BCI-based selection of the Self-medication drug. Step 2: Autonomous navigation to selected shelf. Step 3: Grabbing procedure. Step 4: Drug envelope scanning. Step 5: Robot navigates to patient, in order to deliver the medication. Step 6: Patient approaching. Step 7: Drug delivery.

Figure 12 proposes a MONOCULAR application to a real-life scenario. The proposed case study was realized in the context of the project “Medical Assistance in Contextual awareness” (AMICO) [13]. For the proof of concept, a patient wore a wireless electroencephalography (EEG) headset. A dedicated PC ran a Brain–Computer Interface (BCI) to formalize a self-medication drug request [22,23,24] as shown in Figure 12 panel 1. Once the request was formalized, Pepper autonomously navigated up to the preset repository to recognize and pick up the requested package (Figure 11 panel 2). The robot exploited the MONOCULAR Routine 1 to grab the requested drug (Figure 11 panel 3). Once the grabbing procedure was completed, Pepper scanned the package to find a match with the user’s request (Figure 11 panel 4). In the positive cases, Pepper went back to the patient, holding the pharmaceutical package in a safe position (Figure 11 panels 5 and 6). Next, it helped the user, with audio and video guidelines, to receive the objects, and concluded the delivery (Figure 11 panel 7).

## 4. Conclusions

A novel framework to introduce object manipulation capabilities on social interactive robots mainly designed for verbal and animated interactions has been presented. The proposed framework, named MONOCULAR, is intended to improve social interactive robots’ functionalities by exploiting data from two RGB cameras and a 3D sensor. To generalize MONOCULAR application, this equipment should be preferably embedded in the robot’s head frame to carry out different object manipulation routines. Specifically, two routines have been embedded in the MONOCULAR framework for an object placed both laterally and centrally on a dedicated shelf. The first kind of routine exploits the top RGB camera to stream frames to an online object detection algorithm, the YOLOv3. To realize the grabbing procedure, the YOLOv3 tag is used to align the robot with a specific target; here, a drug package. The 3D sensor is then used to manage the grasping process, evaluating a parameter named hand-vs.-object coverage percentage.

Proper tuning of this parameter demonstrated its capability for ensuring a good success rate in object grabbing, even if no force sensor feedback is present (such as in the commercial Pepper robot).

The MONOCULAR framework was embedded, optimized, characterized, and tested on the Pepper robot by SoftBank Robotics. Experimental results on the target robot demonstrated that the grabbing success rate of a laterally placed object can achieve 96%, even if objects are not positioned or orientated in the nominal position. Thus, a human positioning error does not affect overall grabbing accuracy. The second routine of the MONOCULAR framework concerns centrally placed objects. In this case, the routine mainly exploits the bottom RGB camera and YOLOv3 tags. The proposed routine allows the estimation of the shelf’s height and subsequently manages the arm segments according to the YOLOv3 tag positioning with respect to the camera’s field of view. Experimental results on the Pepper robot showed that grabbing accuracy can achieve a stable 97% for a wide range of different shelf heights, generalizing its applicability.

To conclude, a proof of concept has been realized in the frame of the AMICO Project and presented and discussed here. This proposed case study demonstrated the applicability of the MONOCULAR framework in real-life scenarios, such as drug delivery in an assistive context.

## Data Availability

Not applicable.

## References

[1] Khan Z.H., Sidadique A., Lee C.W. (2020). Robotics Utilization for Healthcare Digitization in Global COVID-19 Management. Int. J. Environ. Res. Public Health.

[2] Yen P.Y., Kellye M., Lopetegui M., Saha A., Loversidge J., Chipps E.M., Gallagher-Ford L., Buck J. (2018). Nurses’ time allocation and multitasking of nursing activities: A time motion study. AMIA Annual Symposium Proceedings.

[3] Fragapane G., Hvolby H.H., Sgarbossa F., Strandhagen J.O. (2020). Autonomous Mobile Robots in Hospital Logistics. Proceedings of the IFIP International Conference on Advances in Production Management Systems.

[4] Lestingi L., Askarpour M., Bersani M.M., Rossi M. (2020). Formal verification of human-robot interaction in healthcare scenarios. Proceedings of the International Conference on Software Engineering and Formal Methods.

[5] MIR Homepage. https://www.mobile-industrial-robots.com/en/insights/case-studies/.

[6] Yumi Homepage. https://new.abb.com/news/detail/37301/abb-demonstrates-concept-of-mobile-laboratory-robot-for-hospital-of-the-future.

[7] UVD Robots Homepage. http://www.uvd-robots.com/.

[8] Ackerman E. (2018). Moxi prototype from diligent robotics starts helping out in hospitals. IEEE Spectrum.

[9] Pfeiffer S., Angulo C. (2015). Gesture learning and execution in a humanoid robot via dynamic movement primitives. Pattern Recognit. Lett..

[10] Dieber B., Breiling B., Taurer S., Kacianka S., Rass S., Schartner P. (2017). Security for the robot operating system. Robot. Auton. Syst..

[11] Pandey A.K., Gelin R. (2018). A mass-produced sociable humanoid robot: Pepper: The first machine of its kind. IEEE Robot. Autom. Mag..

[12] Ikeuchi T., Sakurai R., Furuta K., Kasahara Y., Imamura Y., Shinkai S. (2018). Utilizing social robot to reduce workload of healthcare professionals in psychiatric hospital: A preliminary study. Innov. Aging.

[13] Di Palma V., De Venuto D., Ricci S., Frangi A., Savoia A.S., Di Nocera D., Zampognaro P., Coronato A., Infantino I., Pescosolido L. Medical Assistance in Contextual awareness” (AMICO): A project for a better cardiopathic patients quality of care. Proceedings of the 2019 IEEE 8th International Workshop on Advances in Sensors and Interfaces (IWASI).

[14] Redmon J., Farhadi A. (2018). Yolov3: An incremental improvement. arXiv.

[15] Mao Q.-C., Sun H.M., Liu Y.B., Jia R.S. (2019). Mini-YOLOv3: Real-time object detector for embedded applications. IEEE Access.

[16] Mezzina G., De Venuto D. RGB and 3D-Segmentation Data Combination for the Autonomous Object Manipulation in Personal Care Robotics. Proceedings of the 2021 16th International Conference on Design & Technology of Integrated Systems in Nanoscale Era (DTIS).

[17] Han B.-G., Lee J.-G., Lim K.-T., Choi D.-H. (2020). Design of a Scalable and Fast YOLO for Edge-Computing Devices. Sensors.

[18] Shrivakshan G.T., Chandrasekar C. (2012). A comparison of various edge detection techniques used in image processing. Int. J. Comput. Sci. Issues.

[19] Swami K., Deshpande P.P., Khandelwal G., Vijayvargiya A. Why my photos look sideways or upside down? Detecting canonical orientation of images using convolutional neural networks. Proceedings of the 2017 IEEE International Conference on Multimedia & Expo Workshops (ICMEW).

[20] Borenstein J., Liqiang F. (1996). Measurement and correction of systematic odometry errors in mobile robots. IEEE Trans. Robot. Autom..

[21] De Venuto D., Tio Castro D., Ponomarev Y., Stikvoort E. (2009). Low power 12-bit SAR ADC for autonomous wireless sensors network interface. Proceedings of the 2009 3rd International Workshop on Advances in sensors and Interfaces (IWASI).

[22] De Venuto D.V., Annese F., Mezzina G., Ruta M., Di Sciascio E. Brain-computer interface using P300: A gaming approach for neurocognitive impairment diagnosis. Proceedings of the High Level Design Validation and Test Workshop (HLDVT).

[23] Mezzina G., De Venuto D. Local binary patterning approach for movement related potentials based brain computer interface. Proceedings of the 2019 IEEE 8th International Workshop on Advances in Sensors and Interfaces (IWASI).

[24] Blagojevic M., Kayal M., Gervais M., De Venuto D. (2006). SOI Hall-Sensor Front End for Energy Measurement. IEEE Sens. J..

